# A Call for International Research on COVID Induced Brain Disorders

**DOI:** 10.1093/nsr/nwab190

**Published:** 2021-10-27

**Authors:** Pedro A Valdes-Sosa, Alan C Evans, Mitchell J Valdes-Sosa, Mu-ming Poo

**Affiliations:** Global Brain Consortium, University of Electronic Sciences and Technology of China (UESTC)Chengdu China, Cuban Neurosciences Center (CNEURO), Cuban Academy of Sciences, La Habana Cuba; Global Brain Consortium, Royal Society of Canada, McGill Center for Integrative Neuroscience, McGill University, Montreal, Canada; Cuban Neuroscience Center (CNEURO), Cuban Academy of Sciences, La Habana, Cuba; Center for Excellence in Brain Science and Intelligence Technology, Institute of Neuroscience, Chinese Academy of Sciences, Shanghai, China

## Abstract

COVID-19-Induced Brain Disorders (CIBD) will strain world health systems, complicated by the heterogeneity of manifestations—higher than any other aspect of human biology. Neural, psychological, and social causes must be disentangled for effective population-level management of CIBD. This requires international cooperation leveraging open science and neurotechnologies appropriate for health systems.

## Text

The COVID-19 pandemic, still raging worldwide, imposes staggering human and economic costs. These will only increase as many convalescent patients suffer consequences for many years. Though the virus could target multiple organ systems, we focus on COVID-19-induced brain dysfunction (CIBD), a condition of current concern after its prevalence and consequences have become evident [1][2]. There is evidence that CIBD exhibits extreme variability of susceptibility, physiopathological mechanisms, and brain systems affected [4]—a diversity higher than that of any other aspect of human biology. This protean manifestation is due to the complexity of the brain and the convergence of neural, psychological, and social causal mechanisms. The lack of cost/effective neuro-diagnstic technologies hinders population-level management of CIBD.

Recent studies of electronic records show that six months after recovery, a substantial proportion of retrospectively studied COVID-19 patients had CIBD [3]. Besides direct CIBD resulting from SARS-CoV-2 infection, prolonged disease-associated and work-related stress, social distancing, and quarantine may affect the mental health of large populations of infected and uninfected individuals. Therefore, the math is simple: the COVID-19 contribution to the Global Burden of Brain Disorders might soon compete with other causes. The management of this Burden will be complex. How to screen, identify, and subtype this condition? How to target cost/effective treatment? Seldom before has there been such a need for large-scale personalized medicine. The sheer prevalence and complexity of CIBD seem to distance equitable universal health care. Brain research priorities have to face up to this reality and provide solutions.

ORIGINAL UNEDITED MANUSCRIPT

Lack of clinical and basic knowledge is one dimension of the problem (Figure). Mechanistic modeling must parse empirical associations for evidence-based management of CIBD disease progression. This research may help unravel the twisted skein of CIBD manifestations. The predisposition of people with brain disorders to contract COVID further complicates population studies. As achieved in the UK Biobank, large-scale deep genomic-neuroimaging phenotyping may provide keys to such casual modeling [5]. Smaller, more focused projects, such as the 3T/7T NeNeSCo longitudinal study [6], will be vital to understand physiopathology, for example, the breakdown of the blood-brain barrier.

However, as things stand, this type of research will not significantly reduce the impact of CIBD on the world population. There are several reasons for this:

- The brain imaging modalities that underpin these projects, essential for studying mechanisms, are expensive, have low throughput, and are scarce in economically disadvantaged settings. Thus, they are not suitable for population-based health management.
- Even if scalable diagnostic neurotechnology should become available, a thorny and unsolved problem is the need for increased integration of research into the intersection of brain research and health delivery.

The failure of brain research to reduce the Global Burden of Brain Disorders is not a new problem. The COVID-19 pandemic has only made solving this disconnect more urgent. The WHO/international Brain Projects meeting on Global Brain Health charted out a roadmap to address this problem in Geneva on June 15, 2016. We identified the need to:

1. Create inexpensive and scalable diagnostic neurotechnologies useful in public health settings, regardless of the economic background, and integrate these instruments into projects based on more sophisticated technologies to create translational bridges to health delivery.
2. Develop health models that empower equitable Universal Brain Health care. These must be sustainable and cost-effective. This research in Lower and Middle-Income Countries (LMIC) must be "by" and not "for" the scientific community in these countries. The vaunted "democratization" of data and analytics from the international Brain Projects must be truly made accessible to all.
3. Harness information and communication technologies provide comprehensive coverage, efficient management, and expert consultation at a distance. In addition, developments in artificial intelligence would allow achieving high throughput of diagnostic technologies.

At the meeting, proofs of concept were provided. The Cuban Neuroscience Center argued for quantitative Electroencephalography (qEEG) as a diagnostic translational bridge. We showed that a health delivery model interfacing qEEG and primary health system care is valid for economically limited scenarios. The Global Brain Consortium has pursued these goals developing multi-national norms and for quantitative EEG [7] and demonstrated its use in identifying the effect of early adversity over the lifespan[8].

These results correspond to an increased pace in brain research results across the field, with multimodal data of brain functions gathered at a staggering rate in projects such as the Human Brain Project (HBP), Human Connectome Project (HCP), UK Biobank, and others. The GBC provides access to analytics and data with minimal cost in bandwidth to researchers worldwide, based on the LORIS database system and the CBRAIN portal [9]. The China Brain

ORIGINAL UNEDITED MANUSCRIPT

Project, initiated this year, has specific programs aiming at large-scale diagnostic and intervention studies on brain disorders and data platforms, with the goal of international collaborations in data collection and analysis and data sharing [10]. This framework may be applied to CIBD, as shown by the Cuban study of survivors of COVID-19 showing a differential qEEG signature compared to controls. This signature is being integrated with cognitive tests in a diagnostic toolbox to facilitate triage of CIBD.

The stage is set to work to benefit those afflicted by CIBD. Funding agencies and research institutions must seize the moment and the opportunity and forge the link between research and public health. Ameliorating the worsening of the Global Burden of Brain Disorders due to the pandemic requires research that transcends passive accumulation of knowledge but instead requires completing, in a short time, the gap towards translation to public health settings. In addition, wealthier nations should implement economic models to benefit LMIC. An example of such a program is the Belt and Road Initiative of China.

This perspective is a call to action now. CIBD and the more general problem of Global Brain Health pertinence is not a transitory concern. On the contrary, it is essential to alleviate growing inequities in a world where aging and pandemics are an increasing risk. The concerted efforts to eradicate the pandemic may be providing a roadmap to shorten the trajectory from research to the improvement of people's lives globally.

ORIGINAL UNEDITED M
